# Association of esketamine exposure with secondary sclerosing cholangitis in critically ill patients: a retrospective cohort analysis of 20,000 ICU cases

**DOI:** 10.1186/s40560-026-00906-2

**Published:** 2026-07-08

**Authors:** Sebastian Zeiner, Julian Rettenbacher, Nikolaus Michael Nowak, Andreas Duma, Eva Schaden, Fabian Peter Hammerle, Daniel Laxar, Dieter Adelmann, Johannes Müller, Peter Wohlrab, Oliver Kimberger

**Affiliations:** 1 Division of General Anesthesia and Intensive Care Medicine, Intensive Care Medicine and Pain Medicine, Department of Anesthesia, Medical University of Vienna, Währinger Gürtel 18-20, 1090 Vienna, Austria; 2 Ludwig Boltzmann Institute Digital Health and Patient Safety, Währinger Straße. 104/10, 1180 Vienna, Austria; 3 Department of Anesthesia & Perioperative Care, University of California San Francisco, 521 Parnassus Avenue, Floor 04, Box 0648, San Francisco, CA 94143-0648 USA; 4 Department of Biomedical Imaging and Image-Guided Therapy, Medical University of Vienna, Währinger Gürtel 18-20, 1090 Vienna, Austria; 5 Department of Anesthesiology and Intensive Care Medicine, University Hospital Tulln-Klosterneuburg, Alter Ziegelweg 10, 3430 Tulln an der Donau, Austria; 6 Division of Cardiothoracic Anesthesia and Intensive Care Medicine, Department of Anesthesiology, General Intensive Care and Pain Medicine, Medical University of Vienna and Vienna General Hospital,, Währinger Gürtel 18-20, 1090 Vienna, Austria

**Keywords:** Secondary sclerosing cholangitis, Ketamine, ICU, COVID-19, Cholestasis, Liver injury, Sedation, Risk factors

## Abstract

**Background:**

Secondary sclerosing cholangitis in critically ill patients (SSC-CIP) is a severe cholangiopathy with high mortality observed in ICU survivors. While prolonged mechanical ventilation and COVID-19-associated acute respiratory distress syndrome (ARDS) are established risk factors, the impact of ketamine, a commonly used sedative, remains controversial.

**Methods:**

This retrospective study included 20,973 ICU patients admitted between January 2014 and February 2022 at a tertiary university hospital. Patients with severe cholestasis were preselected (*n* = 532) and reviewed for SSC-CIP diagnosis or likelihood of SSC-CIP. Multivariate logistic regression was used to evaluate Esketamine exposure and dose dependency while controlling for other risk factors.

**Results:**

SSC-CIP was confirmed in 0.11% of patients; 0.20% had clinical profiles compatible with undiagnosed SSC-CIP. Among COVID-19 ICU patients, the incidence was 3.24%. Esketamine administration was significantly associated with SSC-CIP (70% of SSC cases vs. 7% controls). In a multivariate analysis, cumulative Esketamine dose (OR 1.021 per gram), ICU duration (OR 1.031/day), and COVID-19 status (OR 20.6) were independent predictors.

**Conclusion:**

Esketamine exposure was associated with SSC-CIP in a dose-dependent manner, highlighting the need for judicious sedative use in critically ill patients. Our cholestasis-first screening strategy also demonstrates a novel approach for detecting previously unrecognized SSC-CIP cases. Future prospective trials should evaluate the benefits of ketamine-free sedation strategies in high-risk populations.

## Introduction

Secondary sclerosing cholangitis in critically ill patients (SSC-CIP) is a rare but life-threatening liver disease characterized by progressive cholestatic injury leading to biliary destruction and cirrhosis [1]. First described in the early 2000s, SSC-CIP remains underdiagnosed due to its delayed onset, overlapping symptoms with critical illness cholestasis, and diagnostic complexity [2, 3]. Early recognition is vital, as therapeutic options are limited and liver transplantation is often the only curative intervention [3].

While ischemia-induced biliary epithelial damage is the prevailing pathogenic model, risk factors remain incompletely understood [3]. Studies have also identified low mean arterial pressure, prolonged mechanical ventilation, and prone positioning as contributing factors [3–5]. In recent years, SSC-CIP incidence has dramatically increased among COVID-19 ARDS [6] patients, prompting investigations into novel mechanisms, including microvascular dysfunction, cytokine storms, and viral tropism for cholangiocytes [4, 7].

One emerging area of interest is the role of sedatives in hepatobiliary complications. Ketamine, an NMDA receptor antagonist, is widely used in ICUs for analgesia and sedation, particularly in patients with high opioid requirements and/or bronchospasm. However, ketamine is also known for its hepatobiliary side effects in chronic recreational users, ranging from bile duct dilatation to beading and cholestatic liver injury [8–10]. Despite the prevalence of ketamine use in critical care, its contribution to SSC-CIP has received limited systematic evaluation.

In this retrospective cohort study of over 20,000 ICU patients at the Medical University of Vienna, we assessed the incidence of SSC-CIP and its association with Esketamine. We hypothesized that Esketamine use, particularly at high cumulative doses, is independently associated with SSC-CIP development, even when controlling other factors such as COVID-19 and prolonged mechanical ventilation.

## Methods

### 2.1. Study design and setting

We conducted a retrospective cohort study of critically ill patients admitted to the Department of Anesthesiology and Intensive Care Medicine at the Medical University of Vienna between January 1, 2014, and February 28, 2022. The study was approved by the institutional ethics committee (EK Nr: 1185/2022).

### 2.2. Patient population

All patients ≥ 18 years admitted to the ICU during the study period were screened (*N* = 20,980).

### 2.3. SSC-CIP diagnosis

Patients with laboratory signs of cholestasis were identified based on peak serum levels of alkaline phosphatase (ALP ≥ 3 × ULN), gamma-glutamyltransferase (GGT ≥ 3 × ULN), and total bilirubin (> 3 mg/dL). This yielded a cohort of 532 patients for detailed clinical chart review. This yielded a cohort of 532 patients for detailed clinical chart review. These cases were evaluated through a structured chart review process using predefined criteria, including laboratory trends, clinical context, and available diagnostic documentation. All cases were initially reviewed and classified by a single investigator. In cases of ambiguity, as well as for patients classified as “SSC Likely,” a second investigator reviewed the case, and final classification was determined by consensus.

Where ambiguity existed, cases were classified conservatively, meaning that only patients with a high likelihood of SSC-CIP based on consistent laboratory patterns and clinical context were retained, while uncertain cases were preferentially assigned to the non-SSC category to minimize false-positive classification. The classification process was not formally blinded to exposure status due to the retrospective nature of the dataset. However, assessment of esketamine exposure was performed only after completion of the SSC-CIP classification, reducing the risk of bias in case adjudication.

Cases were categorized into three groups:

- Confirmed SSC-CIP: Radiologic or endoscopic findings consistent with bile duct strictures, beading, or vanishing bile ducts, after exclusion of primary sclerosing cholangitis or other chronic liver diseases.
- SSC Likely: Persistent cholestasis as defined above, with no documented normalization of cholestatic laboratory parameters at any time during the ICU stay, consistent with the progressive and typically irreversible nature of SSC-CIP. In addition, cases were required to have no clearly identifiable alternative cause of cholestasis, such as biliary obstruction, acute ischemic hepatitis, or documented drug-induced liver injury. While confirmatory imaging consistent with SSC-CIP was not available in this group, imaging was frequently performed to exclude alternative etiologies, particularly biliary obstruction.
- Non-SSC-CIP: All other cholestatic patients not fulfilling the above criteria, as well as all non-cholestatic patients.

### Exposure variables

Detailed data were collected from electronic medical records, including age, sex, ICU length of stay, duration of mechanical ventilation, main diagnosis of ARDS, COVID-19 status, and cumulative Esketamine dose.

Esketamine administration was recorded as:

Binary exposure (yes/no).

Cumulative dose (in grams).

Duration of use (days).

### 2.4. Statistical analysis

Descriptive statistics were reported as mean ± SD or median [IQR] as appropriate. Univariate analysis was conducted using the Fisher–Freeman–Halton test (categorical variables) and ANOVA (continuous variables). Multivariate logistic regression was used to assess factors associated with SSC-CIP. Statistical significance was set at *p* < 0.05. To account for potential confounding by indication related to esketamine use, a stratified modeling approach was applied. One model was applied to the overall ICU population to evaluate general predictors of SSC-CIP (e.g., ICU length of stay, COVID-19), and a separate model was constructed for the subgroup of patients who received esketamine to assess dose-related association within a more clinically homogeneous cohort. Backward stepwise elimination was used to refine each model retaining only variables that remained independently associated with the outcome in the final model.

## Results

### Study population and classification of SSC-CIP status

A total of 20,973 ICU stays at the University Hospital Vienna between January 2014 and February 2022 were analyzed, after excluding 7 patients with incomplete data or a pre-existing diagnosis of SSC. 532 patients were identified through laboratory-based preselection for severe cholestasis, defined as simultaneous elevations > 3 × upper limit of normal (ULN) in bilirubin, alkaline phosphatase (ALP), and gamma-glutamyl transferase (GGT).

Through a detailed chart review, patients were stratified into three groups:

SSC-CIP (*n* = 23): Diagnosis confirmed by imaging (ERCP, MRCP, or a description of typical findings such as casts and beading).

SSC Likely (*n* = 42): Fulfilled predefined clinical and laboratory criteria for SSC-CIP, including persistent cholestasis without normalization and absence of identifiable alternative causes, but without confirmatory imaging.

No SSC (*n* = 20,908): Remaining patients without clinical suspicion or evidence of SSC.

**Fig. 1:**
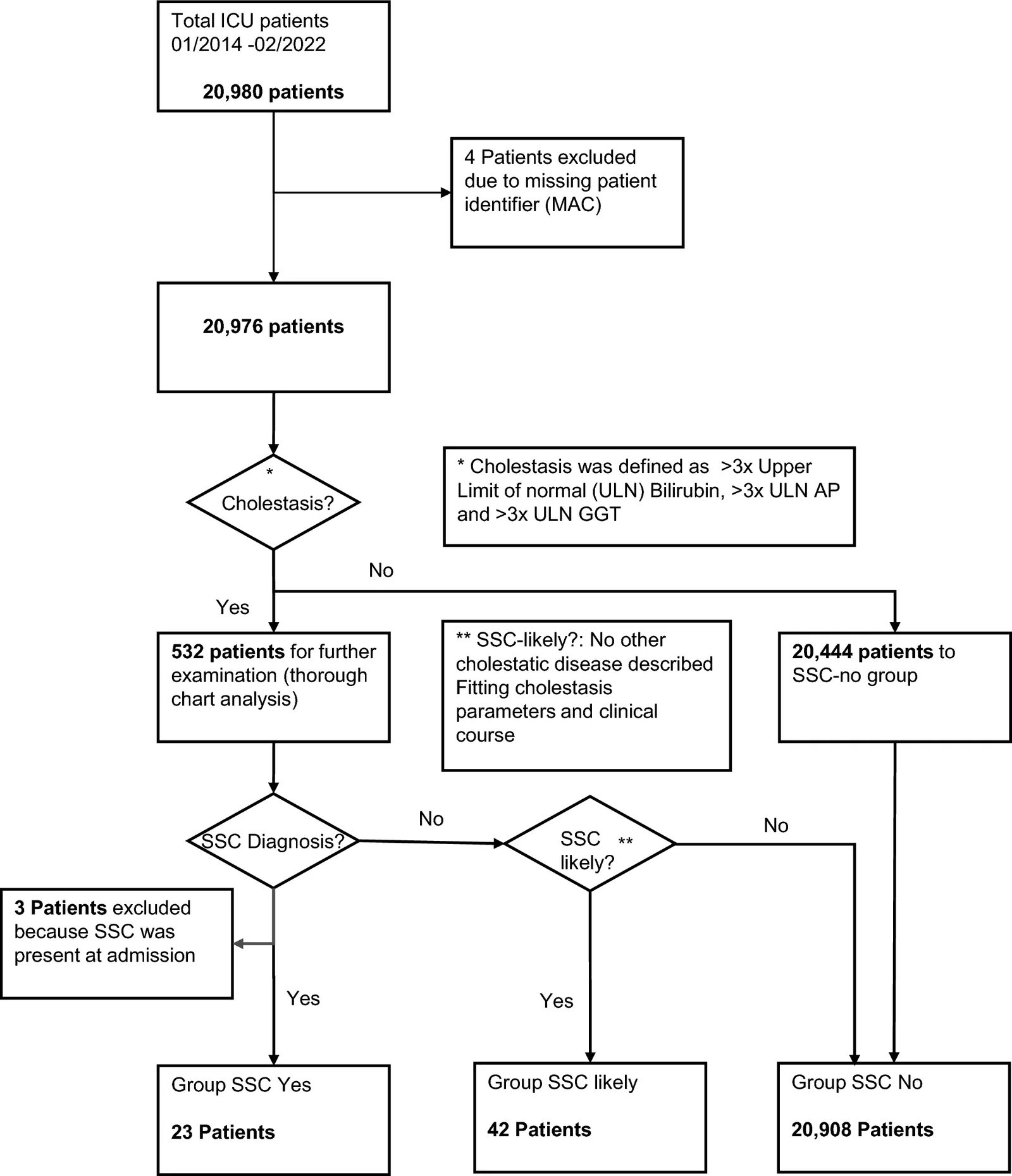
Patient flow chart

The combined “SSC-affected” group (SSC-CIP + SSC Likely) comprised 65 patients. See also Fig. 1 Patient Flow diagram.

### Incidence of SSC-CIP

The incidence of confirmed SSC-CIP was 0.11% (95% CI 0.065–0.15%). When including patients in the SSC Likely group, the overall estimated burden of SSC-CIP or possible undiagnosed SSC-CIP rose to 0.31% (95% CI 0.24–0.38%).

Among the 185 patients with a primary diagnosis of COVID-19, 6 cases of SSC-CIP were identified, corresponding to a significantly elevated incidence of 3.24% (95% CI 0.68–5.79%). This incidence was more than 30 times higher than the non-COVID cohort (0.09%, *p* < 0.001, Fisher’s exact test). When SSC Likely cases were included, 7.57% (95% CI 3.75–11.38%) of COVID-19 patients met criteria for SSC-CIP or probable SSC.

### Esketamine exposure across groups

Esketamine was administered to 1,449 patients (6.9%), the majority of whom had prolonged ICU stays. Among confirmed SSC-CIP patients, 70% received esketamine during their ICU stay, compared to only 7% in patients without SSC-CIP. This yielded an SSC-CIP incidence of 1.1% in the esketamine-exposed population.

Within patients receiving Esketamine, patients with confirmed SSC-CIP were characterized by:

- Longer duration of administration: Mean of 21.1 ± 19.6 days vs. 8.0 ± 9.5 days in controls
- Higher cumulative dose: Mean of 39.2 ± 27.8 g vs. 14.2 ± 20.0 g in controls
- The higher mean daily dose was not significantly different between groups; duration of exposure was the primary differentiating factor.

Univariate Analysis of Factors Associated with SSC-CIP:

A univariate analysis of demographic and ICU variables revealed that SSC-CIP patients:

Were more likely to be male (70% vs. 55%, *p* = 0.038), had significantly longer ICU stays (57.4 ± 47.0 days vs. 8.1 ± 13.5 days, *p* < 0.001), were ventilated for longer durations (40.6 ± 38.8 days vs. 5.1 ± 10.8 days, *p* < 0.001), had higher average PEEP values (7.6 ± 1.6 cmH₂O vs. 6.0 ± 1.6 cmH₂O, *p* = 0.015) and had similar BMI compared to the general cohort (*p* = 0.42) (Table 1).

**Table 1:** Univariate exploratory analysis of risk factors

Group	SSC Yes	SSC Likely	SSC No	*p* value	
Gender (% male)	16/23(70%)	32/42 (76%)	11,428/20,908 (55%)	0.006	(1)
ICU stay (mean, days)	57.35 ± 47.03	61.38 ± 52.17	8.12 ± 13.46	< .001	(2)
Age (mean)	55.65 ± 11.44	49.83 ± 14.64	58.54 ± 16.50	0.002	(2)
BMI (mean)	24.49 ± 4.32	27.57 ± 7.31	26.54 ± 6.16	0.193	(3)
Mechanical ventilation received (% yes)	23/23 (100%)	41/42 (98%)	19,829/20,908 (95%)	0.384	(1)
Ventilation (mean, days)	40.65 ± 38.81	48.44 ± 43.54	5.07 ± 10.80	< .001	(2,a)
Avg PEEP (mean, mbar)	7.62 ± 1.58	7.76 ± 1.57	6.05 ± 1.59	< .001	(3,a)
Esketamine (% yes)	16/23 (70%)	28/42 (67%)	1,405/20,908 (7%)	< .001	(1)
Esketamine (mean, days)	21.13 ± 19.55	19.21 ± 14.85	7.95 ± 9.48	< .001	(2,b)
Esketamine cumulative dose (mean, g)	39.17 ± 27,82	33.36 ± 30,42	14.19 ± 20.04	< .001	(2,b)

^1^Exact test (Fisher-Freeman-Halton)

^2^Welch test

^3^ANOVA

^a^Filtered to ventilated patients (19,893)

^b^Filtered to Esketamine receiving patients (1,449)

In the overall ICU cohort (*n* = 20,973), multivariate logistic regression was performed to identify factors associated with SSC-CIP (see Table 3). The initial model included ICU length of stay, mechanical ventilation duration, and COVID-19 diagnosis. After backward elimination, mechanical ventilation duration was removed, and the final model retained ICU length of stay and COVID-19 status. The model demonstrated a good fit (*χ*^2^ = 227.0, *df* = 4, *p* < 0.001) with a Nagelkerke R2 of 0.201, indicating that approximately 20.1% of the variance in SSC-CIP occurrence was explained by the included predictors. ICU length of stay was independently associated with increased odds of SSC-CIP, with an odds ratio (OR) of 1.031 per day. Additionally, a primary diagnosis of COVID-19 was strongly associated with SSC-CIP, with a 20.6-fold increase in odds compared to non-COVID patients (Table 2).

**Table 2:** Multivariate logistic regression for overall cohort

SSC status^a^	*Predictor*	B	SE	*Wald*	df	p-value	Exp(B)	95% confidence interval Exp(B)	
								Lower limit	Upper limit
SSC Yes	Constant term	− 7.628	0.269	804.745	1	< 0.001			
	ICU stay length	0.030	0.003	101.922	1	< 0.001	1.031	1.025	1.037
	Covid Status	3.025	0.467	41.953	1	< 0.001	20.598	8.246	51.451
SSC Likely	Constant term	− 6.945	0.194	1284.713	1	< 0.001			
	ICU stay length	0.031	0.002	174.443	1	< 0.001	1.032	1.027	1.036
	Covid Status	2.431	0.422	33.253	1	< 0.001	11.373	4.977	25.986

^a^The reference category was SSC No

In the subgroup of patients who received Esketamine (*n* = 1,449), a multivariate logistic regression model was used to identify factors associated with SSC-CIP (see Table 3). The model included ICU length of stay, mechanical ventilation duration, cumulative esketamine dose (in grams), duration of esketamine use, and COVID-19 diagnosis. After backward elimination, mechanical ventilation days and Esketamine duration were excluded. The final model demonstrated good fit (*χ*^2^ = 90.1, *df* = 6, *p* < 0.001) and explained 22.5% of the variance. Three variables remained significant: ICU length of stay (odds ratio [OR] 1.029 per day, *p* = 0.006), cumulative esketamine dose (OR 1.021 per gram, *p* = 0.002), and COVID-19 diagnosis (OR 4.13, *p* = 0.047). These findings indicate that in patients receiving esketamine, both longer ICU stay and higher esketamine dose were associated with higher odds of SSC-CIP, with the magnitude of the association for esketamine dose comparable to that of each additional ICU day (Table 3).

**Table 3:** Multivariate logistic regression for esketamine patients

		*B*	SE	Wald	*df*	p-value	Exp(B)	95% confidence interval Exp(B)	
								Lower limit	Upper limit
SSC Yes	Constant term	− 6.602	0.555	141.652	1	0.000			
	ICU stay length	0.029	0.005	30.861	1	0.000	1.029	1.019	1.040
	Esketamine (g)	0.021	0.009	6.047	1	0.014	1.021	1.004	1.038
	Covid status	1.416	0.568	6.219	1	0.013	4.119	1.354	12.531
SSC Likely	Constant term	− 5.627	0.385	213.159	1	0.000			
	ICU stay length	0.027	0.004	40.480	1	0.000	1.028	1.019	1.036
	Esketamine (g)	0.017	0.007	6.219	1	0.013	1.017	1.004	1.031
	Covid status	0.879	0.482	3.321	1	0.068	2.408	0.936	6.194

Patient characteristics—Esketamine Group		
	Mean	Std. deviation
Total patients	1,449	–
Gender (male) (m%)	901 (62%)	–
Age (years)	50.55	16.04
BMI	27.75	7.11
ICU stay (days)	28.31	26.55

^a^The reference category was SSC No

## Discussion

In this retrospective cohort study of over 20,000 ICU admissions, esketamine exposure was associated with SSC-CIP in a dose-dependent manner in this large retrospective ICU cohort. The incidence of SSC-CIP in the overall ICU population was 0.11%. This rate aligns with previously reported incidences in ICU populations ranging between 0.05 and 0.14% [1, 7, 11]. Among patients with COVID-19 ARDS, a significantly higher incidence of 3.24% was found, comparable incidences for COVID-19 ARDS patients have been reported in the literature [6, 14]. A dose-dependent relationship between cumulative esketamine use and SSC-CIP was observed, even after adjusting for COVID-19 status and ICU length of stay. These findings suggest that esketamine exposure may represent a potentially modifiable factor, although causal interpretation is limited by the observational design.

### 4.1. Dose response

The most striking finding of this study is the strong association between cumulative Esketamine dose and SSC-CIP. We observed a linear dose–response relationship between Esketamine and SSC-CIP risk with an adjusted odds ratio of 1.021 per gram. Although the per gram effect size appears small, its multiplicative nature results in clinically relevant differences at higher cumulative doses. For example, cumulative exposures of 10 g, 20 g, and 30 g correspond to approximate odds ratios of 1.23, 1.49, and 1.81, respectively. This highlights that patients with prolonged ICU courses and higher cumulative exposure may experience substantially increased relative odds of SSC-CIP. At the observed mean daily Esketamine dose of approximately 1.78 g, this corresponds to an estimated increase in odds of approximately 3.8% per day (OR ≈ 1.038), further illustrating how prolonged exposure over time may translate into clinically meaningful differences in risk. Esketamine is not administered randomly but is typically used in patients with severe respiratory failure, prolonged mechanical ventilation, and high sedation requirements. Therefore, cumulative esketamine exposure may primarily act as a surrogate marker of underlying illness severity rather than a direct causal contributor to SSC-CIP. The absence of key severity indicators limits adjustment for confounding and precludes causal inference. Accordingly, all findings should be interpreted as associative rather than causal.

These results are consistent with the previously published data on both ICU-based Esketamine administration and recreational Esketamine abuse. Among COVID-19 patients, Leonhardt et al. reported significantly higher Esketamine use in SSC-CIP patients (96%) when compared with controls (68%) [6]. Wendel-Garcia et al. also identified a correlation between Esketamine dose and cholestatic liver injury, independent of other sedatives [12]. Outside the ICU, MR cholangiography in chronic Esketamine users has shown bile duct strictures and “beading” patterns resembling SSC, with partial reversibility upon abstinence [13]. The cumulative IV doses in ICU patients are orders of magnitude higher, making direct toxicity or flow impairment at the sphincter of Oddi a plausible mechanistic contributor.

### 4.2. COVID-19 and SSC-CIP: Amplifier, Confounder, or Co-conspirator?

The incidence of SSC-CIP among COVID-19 ARDS patients in our cohort was 3.24%, over 30 times higher than in non-COVID patients. This finding aligns with prior studies reporting SSC-CIP rates between 0.6 and 11.7% in similar populations [5, 14–16]. Although SARS-CoV-2 may directly affect cholangiocytes via ACE2 receptors [17], the pathogenesis is likely multifactorial. In our analysis, COVID-19 remained associated with higher odds of SSC-CIP, even after adjusting for Esketamine exposure and ICU duration. Whether this reflects virus-specific tropism, immune dysregulation, or treatment-related ischemia remains unclear.

Importantly, Esketamine use was particularly high in COVID-19 ARDS management, driven by sedation challenges during prolonged prone ventilation, high ventilatory pressures, and ECMO. This raises the possibility that Esketamine and COVID-19 may have synergistic effects, and that Esketamine’s hepatobiliary risks are amplified in the COVID-19 inflammatory milieu. In addition, COVID-19 status was defined based on the primary diagnosis coding, and patients with concomitant SARS-CoV-2 infection during ICU stay may not have been fully captured. This may have resulted in misclassification and underestimation of COVID-19 exposure.

### 4.3. Underdiagnosis and the utility of laboratory-based screening

The identification of 40 additional cases classified as “probable SSC-CIP” supports the hypothesis that SSC-CIP remains underdiagnosed. These patients showed persistent cholestasis, prolonged ICU courses, and similar patterns of Esketamine exposure as confirmed SSC cases. Prior literature suggests that up to 50% of SSC-CIP patients may die before diagnosis [18]and our findings corroborate this by identifying a clinically indistinguishable SSC-like group based on chart review and laboratory results. Our strategy of preselecting patients using cholestatic lab thresholds (ALP ≥ 3 × ULN, GGT ≥ 3 × ULN, bilirubin > 3 × ULN) proved effective at identifying high-risk patients. It also enabled estimation of a broader SSC-CIP burden (0.31%) and could be used prospectively to trigger early imaging or hepatology referral. In contrast to the standard approach of working backward from ERCP databases or ICD-10 codes, we identified SSC-CIP cases by systematically screening for cholestasis first and then performing a detailed chart review. By analyzing more than 500 patient records, we defined an SSC Likely group comprising patients without a formal SSC diagnosis who nonetheless had no alternative explanation for cholestasis and demonstrated clinical features consistent with SSC-CIP. Importantly, patients in the SSC Likely group demonstrated persistently elevated cholestatic laboratory values without normalization throughout their ICU stay, reflecting the progressive and typically irreversible nature of SSC-CIP. In addition, the overall pattern and magnitude of laboratory abnormalities in this group were clinically comparable to those observed in patients with confirmed SSC-CIP, further supporting the biological plausibility of this classification despite the absence of confirmatory imaging.

This approach introduces a methodological framework that has not previously been applied in SSC-CIP research.

### 4.4. Implications for clinical practice

Esketamine exposure remained associated with SSC-CIP after multivariable adjustment, and the relationship was dose-dependent. As esketamine use increases in ICUs—particularly in COVID-19, burn injury, or ECMO contexts- clinicians should be aware of its potential hepatobiliary toxicity. This is further supported by recent French data showing improved survival and lower rates of cholestatic liver injury after ketamine-restrictive protocols in burn ICUs, where ketamine is limited to very low, capped doses as second-line analgesia rather than used for high-dose continuous sedation [19].

While ketamine remains a valuable sedative, especially in difficult-to-sedate patients or when minimizing opioid and GABAergic burden, its routine use in patients with signs of early cholestasis or multiple risk factors for SSC-CIP may warrant reconsideration. Alternative strategies (e.g., rotating sedatives, early imaging in high-risk cases) should be explored.

### 4.5. Limitations

This study is subject to the inherent limitations of retrospective designs. Although esketamine exposure was associated with SSC-CIP after multivariable adjustment, including ICU length of stay and mechanical ventilation duration, we cannot exclude residual confounding, particularly from variables related to illness (e.g., SOFA/APACHE scores), vasopressor use, ECMO support, or hepatic perfusion indices, which were not available. COVID-19 status was only captured if it was the primary diagnosis, potentially underestimating actual exposure. Esketamine is not administered randomly but is typically used in patients with severe respiratory failure, prolonged mechanical ventilation, and high sedation requirements. Therefore, cumulative esketamine exposure may primarily reflect underlying illness severity rather than a direct causal effect. The absence of key severity indicators limits adjustment for confounding and precludes causal inference. Accordingly, all findings should be interpreted as associative rather than causal.

During ICU admission, laboratory parameters are typically monitored on a daily basis, increasing the likelihood of detecting cholestatic abnormalities. Patients with prolonged ICU stays are therefore more likely to undergo repeated testing and diagnostic evaluation, increasing the probability of SSC-CIP detection. At the same time, patients transferred earlier to lower-acuity settings may undergo less frequent laboratory monitoring, potentially leading to underdetection of evolving cholestasis. Given that cumulative esketamine exposure is closely linked to ICU length of stay, this differential detection may contribute to the observed association between esketamine use and SSC-CIP.

Another limitation is the reliance on electronic records and imaging reports for SSC-CIP classification. Future studies incorporating bile cultures, imaging review, and histology could improve diagnostic accuracy. Although alternative causes were actively excluded during chart review, incomplete documentation and overlapping pathophysiology in critically ill patients may still result in misclassification. The inclusion of an ‘SSC Likely’ category, while improving sensitivity, inherently trades specificity for sensitivity, which should be kept in mind when interpreting the analysis.

This study reflects a single-center experience, and sedation practices, diagnostic strategies, and patient populations may differ across institutions, limiting generalizability. In addition, sedation strategies and the use of adjunct agents such as esketamine may vary across institutions, healthcare systems, and geographic regions, reflecting evolving ICU sedation paradigms favoring multimodal and opioid-sparing strategies. Current international ICU sedation guidelines (e.g., SCCM PADIS and ESICM recommendations) emphasize light sedation and multimodal approaches but do not provide specific guidance on the use of esketamine, which may contribute to variability in clinical practice. This variability may further limit the generalizability of our findings. [20, 21]

### 4.6. Conclusions

Esketamine exposure was associated with SSC-CIP in a dose-dependent manner in critically ill patients. In the context of COVID-19 and prolonged ICU care, this association may be amplified. Our findings support the growing concern over esketamine’s hepatobiliary safety profile and suggest the need for cautious use, especially in patients with multiple SSC-CIP risk factors or early cholestasis.

## Data Availability

The data that support the findings of this study are available from the corresponding author upon reasonable request and with permission of the relevant ethics committee.

## Declarations

### Generative AI

During the preparation of this work, the authors used ChatGPT Version 5.2 (OpenAI, San Francisco, USA) for assistance with spelling, phrasing, and language refinement. The authors subsequently reviewed and edited all content and take full responsibility for the final version of the manuscript.
